# Alzheimer’s Prediction Methods with Harris Hawks Optimization (HHO) and Deep Learning-Based Approach Using an MLP-LSTM Hybrid Network

**DOI:** 10.3390/diagnostics15030377

**Published:** 2025-02-05

**Authors:** Raheleh Ghadami, Javad Rahebi

**Affiliations:** 1 Department of Computer Engineering, Istanbul Topkapi University, 34662 Istanbul, Türkiye; melisarahebi@topkapi.edu.tr; 2 Department of Software Engineering, Istanbul Topkapi University, 34662 Istanbul, Türkiye

**Keywords:** Alzheimer’s disease, magnetic resonance images (MRI), convolutional neural network (CNN), LSTM neural network, Harris Hawks Optimization (HHO) algorithm

## Abstract

****Background/Objective**:** Alzheimer’s disease is a progressive brain syndrome causing cognitive decline and, ultimately, death. Early diagnosis is essential for timely medical intervention, with MRI medical imaging serving as a primary diagnostic tool. Machine learning (ML) and deep learning (DL) methods are increasingly utilized to analyze these images, but accurately distinguishing between healthy and diseased states remains a challenge. This study aims to address these limitations by developing an integrated approach combining swarm intelligence with ML and DL techniques for Alzheimer’s disease classification. **Method:** This proposal methodology involves sourcing Alzheimer’s disease-related MRI images and extracting features using convolutional neural networks (CNNs) and the Gray Level Co-occurrence Matrix (GLCM). The Harris Hawks Optimization (HHO) algorithm is applied to select the most significant features. The selected features are used to train a multi-layer perceptron (MLP) neural network and further processed using a long short-term (LSTM) memory network in order to classify tumors as malignant or benign. The Alzheimer’s Disease Neuroimaging Initiative (ADNI) dataset is utilized for assessment. **Results:** The proposed method achieved a classification accuracy of 97.59%, sensitivity of 97.41%, and precision of 97.25%, outperforming other models, including VGG16, GLCM, and ResNet-50, in diagnosing Alzheimer’s disease. **Conclusions:** The results demonstrate the efficacy of the proposed approach in enhancing Alzheimer’s disease diagnosis through improved feature extraction and selection techniques. These findings highlight the potential for advanced ML and DL integration to improve diagnostic tools in medical imaging applications.

## 1. Introduction

Dementia impacts a wide range of cognitive processes like reasoning, planning, judgment, or remembering, and is characterized by a decline in these faculties. In older adults, most dementia cases are caused by Alzheimer’s disease. At present, there are 57 million adults suffering from dementia across the globe. Furthermore, the number of Alzheimer’s cases is predicted to reach 152 million, with incidence expected to rise threefold by the year 2050 (Alzheimer’s Disease International (2018). World Alzheimer Report 2018). The underlying cause of such skyrocketing numbers of dementia cases is the growth of the population, which increases the number of people whose age complicates the onset of neurological diseases. The difficulty of diagnosis in neurological diseases arises from the poor sensitivity and specificity of both the structural abnormalities and tests for the diagnosis of Alzheimer’s disease. There is no reliable technique to determine whether pre-dementia will progress to dementia. However, it is risky to run tests to diagnose the neurological condition because they would lead to brain tissue destruction [1].

Alzheimer’s disease is a progressive neurologic disorder with symptoms of memory loss, cognitive impairments, and language difficulties; a cure has not been developed. Early intervention can help retain brain functions by delaying this process. However, there is an issue around the integration of mild cognitive decline and high misdiagnosis rates. Also, accurate neuroimaging is important to ensure better diagnosis and treatment outcomes [2].

The human brain contains roughly 100 billion neurons interacting via synaptic connections to form a complex network with subtle and random processes [3]. EEG offers one excellent, affordable, non-invasive imaging modality that provides high temporal detail in the brain’s electrical activity due to neurotransmission. It is being used more and more in medical diagnostics of Alzheimer’s disease, since all the proofs based on the available literature support its ability to distinguish between neurological features and cognitive disorders more effectively [4]. The onset of Alzheimer’s disease is associated with several pathological changes such as heightened oxidative stress, shrinkage of synapses, intracellular tau accumulation, and buildup of extracellular β-amyloid plaques [5].

Alzheimer’s disease is marked by a myriad of neurological problems resulting in amnesia and degraded functionality of those affected. While many treatments have been proposed, most have proved less effective and full of problems. The disease targets several brain pathways, and therefore, diagnosis can be tricky. However, accumulated evidence from recent studies shows that a flavonoid-rich diet can help maintain health, mental function, and cognitive ability [6]. Some flavonoids inhibit the development of Alzheimer’s disease. Flavonoids, abundant in cocoa, citrus fruits, green tea, and berries, may provide neuroprotection through their action on various intracellular processes that support nerve health and possibly prevent Alzheimer’s disease [7,8].

Physicians use positron emission tomography (PET), MRI, and CT when diagnosing Alzheimer’s disease. PET, in particular, is beneficial for assessing brain lesions’ metabolic and functional state, which assists in early diagnosis. Early diagnosis and treatment of other disorders, such as mild cognitive impairment, make it possible to delay the disease processes [9].

Classifying Alzheimer’s-related images manually by neurologists is time-consuming and often inaccurate. Factors such as the patient’s age, anxiety, and poor vision can further complicate the diagnosis process. Due to the variable presenting symptoms and the difficulty in clinically diagnosing the disease, much research is directed at finding neurobiological signs of the disease associated with early and continuing changes in brain tissue specific to Alzheimer’s disease [10]. Such changes might begin years before obvious clinical presentation [11]. Alzheimer’s disease leads to an abnormal change in the brain, notably amyloid plaques and tau tangles, which progressively destroy healthy neurons and break important neuronal connections [12].

Classification methods such as CNN and LSTM are used for Alzheimer’s diagnosis [13,14]. DL methods also use feature extraction and feature selection methods to reduce Alzheimer’s diagnosis errors [15,16]. In some studies, machine learning methods use DL [17,18,19]. A review of studies in the field of classification of medical images for Alzheimer’s diagnosis shows that these methods face many challenges. An oversight in the existing methods is not using intelligent feature selection methods, especially group intelligence methods [20]. In most studies, DL methods have been used to classify images, but most methods have significant error rates [21]. The proposed method for accurate classification of medical images related to Alzheimer’s disease uses a multi-stage approach based on DL, group intelligence, and machine learning. In the first phase, a CNN is used to extract features. In this first stage, the features discovered by the CNN are combined with the GLCM method [22]. In the second step, the HHO algorithm [23] is applied to feature selection. In the feature selection phase, a multilayer neural network is used to evaluate the attribute vectors. In the third step, the attributes chosen by the LSTM method are delivered to classify images related to Alzheimer’s.

One goal of this article is early and accurate diagnosis of Alzheimer’s disease to save the lives of sick people. Another goal of this research is to reduce the error of DL and machine learning methods in combination with swarm intelligence.

### 1.1. Contribution

Alzheimer’s disease is a neurodegenerative condition characterized by memory loss, cognitive decline, and language difficulties, with no known cure. However, early intervention can slow its progression and help preserve brain function. To improve diagnosis and treatment outcomes, precise neuroimaging and advanced analytical techniques are crucial. This study introduces a novel hybrid framework that integrates multiple advanced methodologies to enhance Alzheimer’s diagnosis.

1. Feature Extraction Integration: The integration of the ResNet-50 and GLCM methods for feature extraction combines high-level DL features with detailed texture analysis, addressing the limitations in using single methods.
  - Objective: To improve the representation of medical images by capturing both global and local features, thereby improving the classification accuracy for Alzheimer’s disease.
2. Optimized Feature Selection: The HHO algorithm is employed to select the most significant features, reducing dimensionality while preserving essential information.
  - Objective: To ensure that only the most relevant features are utilized for classification, minimizing computational burden and optimizing performance.
3. Hybrid Classification Architecture: A combination of an MLP network and an LSTM network is used to classify images into malignant and benign categories.
  - Objective: To use LSTM’s temporal learning capabilities for sequential data, while maintaining high classification performance with MLP.
4. Early Diagnosis Focus: The framework is specifically designed to facilitate the early and accurate diagnosis of Alzheimer’s disease, which is important for timely medical intervention.
  - Objective: To provide a reliable tool for detecting Alzheimer’s at earlier stages, potentially improving patient outcomes.
5. Evaluation and Robustness: The proposed method is evaluated by using the ADNI and MIRIAD datasets, demonstrating superior performance compared to existing methods.
  - Objective: To validate the effectiveness and versatility of the framework through extensive testing and comparison.

Through these contributions, this study offers a comprehensive solution to the challenges of Alzheimer’s diagnosis, advancing methodologies in feature extraction, optimization, and classification.

### 1.2. Organization

This article is organized into several key sections. Section 2 comprehensively reviews related work in Alzheimer’s disease diagnosis, offering insights into existing methodologies. In Section 3, the proposed approach is introduced, combining CNN, HHO, and LSTM networks to enhance the classification of medical images for Alzheimer’s diagnosis. Section 4 details the implementation and evaluation of the proposed method using Python. Finally, Section 5 presents the study’s results and recommendations for future research directions.

## 2. Related Work

Dementia, particularly Alzheimer’s disease (AD), poses a significant global health challenge, with alarming statistics highlighting a rapid increase in prevalence and associated care costs [24]. The financial burden of dementia care reached USD 1 trillion last year, with a notable growth rate observed in low- and middle-income countries [25]. The global cost of dementia care is expected to exceed USD 2 trillion by 2030 [26]. This financial strain is particularly pronounced in low- and middle-income countries, where healthcare systems are less equipped to manage the growing demand [25]. However, recent developments in disease-modifying therapies (DMTs) offer hope, with drugs like lecanemab showing promise in altering disease progression [26,27]. Early diagnosis and intervention are critical, as the effectiveness of DMTs diminishes with delayed treatment [25].

At present, there is no universally effective or definitive strategy for diagnosing or treating Alzheimer’s disease. Nevertheless, early diagnosis is vital in lessening the disease’s effects and improving patients’ quality of life. Traditional diagnostic approaches for Alzheimer’s include mental status evaluations, neurological and physical examinations, and thorough patient history assessments. Laboratory tests like blood, urine, and genetic screenings are also conducted. These tests help to evaluate other conditions that might affect cognitive function, including levels of vitamins and nutrients, infections, and liver, kidney, and thyroid health. Genetic testing is utilized to identify a family history of dementia.

With advancements in medical technology, diagnostic techniques have also evolved. Brain scans, for instance, are utilized to detect Alzheimer’s by examining medical images for signs of brain atrophy or enlargement in certain areas. These scans, in particular, aim at brain regions including the hippocampus and the cerebral cortex since they are responsible for memory, speech, judgment and thinking activities, all of which are significantly altered by the advancement of AD [28].

**Figure 1:**
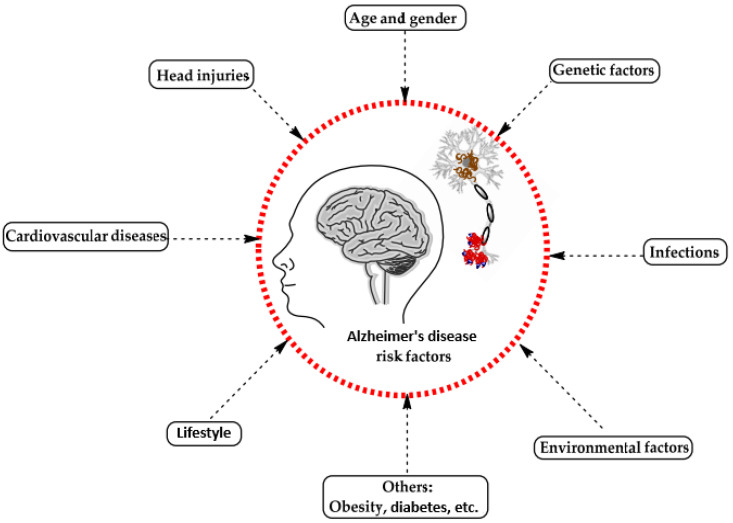
Influences on the development of Alzheimer’s disease [29].

**Figure 2:**
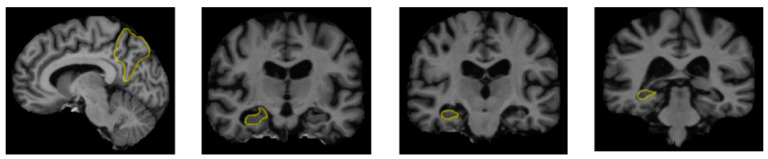
The regions of the brain affected by Alzheimer’s disease in MRI images [30].

A range of influences affect the onset of Alzheimer’s disease, among them lifestyle choices, cardiovascular health, head trauma, age, gender, genetic predispositions, infections, environmental influences, and pre-existing conditions like diabetes, as depicted in Figure 1. In individuals with Alzheimer’s, abnormal protein formations such as amyloid plaques and neurofibrillary tangles develop in and around neurons in specific areas of the brain [29]. These pathological changes lead to the deterioration and damage of brain tissues, as shown in Figure 2 [30].

An effective method for diagnosing Alzheimer’s involves the application of DL and ML approaches. These advanced methodologies are frequently employed to analyze and classify medical images or specific regions within these images, and numerous studies have been conducted using these technologies in regards to Alzheimer’s diagnosis.

In [31], a GAN-based DL approach was proposed for diagnosing Alzheimer’s disease utilizing PET images, achieving an accuracy of about 96.03%. This approach benefits from a DL network grounded in game theory, providing high accuracy with a small amount of training data. However, a notable drawback is the considerable training time required for GAN networks.

In [32], a DL classification technique for MRI images was introduced to detect Alzheimer’s disease plaques, reaching a sensitivity of around 98%. While this method offers high accuracy, its performance relies on a narrow selection of images, which is a significant limitation.

In [33], DL for diagnosing Alzheimer’s disease in MRI images was explored, utilizing VGG19 and DenseNet169 architectures. The study found that these models have strong classification abilities, but a key challenge is the absence of regionalization techniques to distinguish specific brain regions.

In [34], a DL approach employing MRI images was presented to diagnose Alzheimer’s. The research demonstrated that the ResNet framework with multiple layers yielded the best results, though the method lacks a robust feature selection phase.

In [35], a review was conducted on the automatic classification and diagnosis of Alzheimer’s disease employing brain MRI images and DL techniques. This research underscored previous studies that developed precise methods for automatically detecting Alzheimer’s through MRI.

Another study [36] developed a ML technique for classifying and detecting Alzheimer’s disease using retinal vessel images, with an average classification accuracy of around 82.44%. The advantage of this method lies in its use of innovative data, such as retinal images, for diagnosis. However, its relatively low precision in detecting Alzheimer’s is a challenge.

In [37], a DL technique for classifying Alzheimer’s disease using structural magnetic resonance imaging (sMRI) achieved 96% sensitivity and 95% precision by analyzing seven morphological brain characteristics. This method benefits from reduced data dimensionality through feature selection but faces challenges with lengthy training and classification times.

The authors of [38] presented a comprehensive review of DL approaches for Alzheimer’s diagnosis. The findings indicated that, although DL has achieved significant accuracy and performance, these methods face limitations, such as restricted datasets and lengthy training durations.

In [39], a multi-state DL approach was introduced to forecast the progression of Alzheimer’s disease utilizing time series data. This model effectively predicts individuals’ disease progression based on a learning framework.

In [40], a deep neural network incorporating several convolutional layers was conjectured for AD diagnosis via multi-class categorization. Drawing inspiration from the Oxford Net learning model, this network was designed to classify various stages of dementia. The experiments demonstrated that the method achieved an accuracy exceeding 95% for most samples in identifying Alzheimer’s patients.

## 3. Methodology

Combination of ResNet-50 and GLCM Features for Alzheimer’s Diagnosis Disease:

The proposed method for differentiating between malignant and benign brain images in Alzheimer’s disease uses a hybrid architecture that integrates the features of both ResNet-50 and GLCM. The approach includes several key steps that contribute to enhancing model performance and improving the accuracy of MRI image analysis.

1. Complementary Feature Extraction: ResNet-50 is a very important DL architecture capable of extracting high-level features from images. It can identify complex patterns and structures, such as the overall shape and texture of the brain. GLCM is a traditional texture analysis technique that focuses on texture-related features, identifying small differences in aspects such as contrast, homogeneity, and entropy.
2. Improved Information Representation: By combining the features extracted from ResNet-50 and GLCM, the model achieves a richer and more precise representation of MRI images. The high-level features from ResNet-50, along with the texture-based features of GLCM, enable the model to better understand both the structural and textural aspects of the images. This is crucial for the accurate diagnosis of AD.
3. Feature Selection using the HHO Algorithm: After combining the features, the HHO algorithm is used to select the most relevant features from the combined set. HHO helps eliminate redundant or irrelevant features, leaving only the key features critical for Alzheimer’s diagnosis. This process reduces the data dimensions, enhancing both the efficiency and accuracy of the model.
4. Classification using the LSTM Model: Once the optimal features are selected, the LSTM model is trained to classify the images into two categories: benign and malignant. LSTM, known for its ability to capture sequential dependencies, efficiently processes the feature sequences and identifies patterns essential for accurate classification. The integration of optimal features and a robust classification model leads to improved performance.
5. Superior Performance: By integrating both ResNet-50 and GLCM feature extraction methods, the model benefits from both DL and texture-based approaches, resulting in better classification outcomes. The proposed method achieves higher accuracy, sensitivity, and precision compared to other models, such as ResNet-50, VGG16, and traditional GLCM methods. This hybrid approach significantly enhances classification performance, enabling the model to use both DL and texture analysis methods. As a result, it provides superior detection of Alzheimer’s images, leading to better results than single-method approaches.

### 3.1. The Proposed Framework

**Figure 3:**
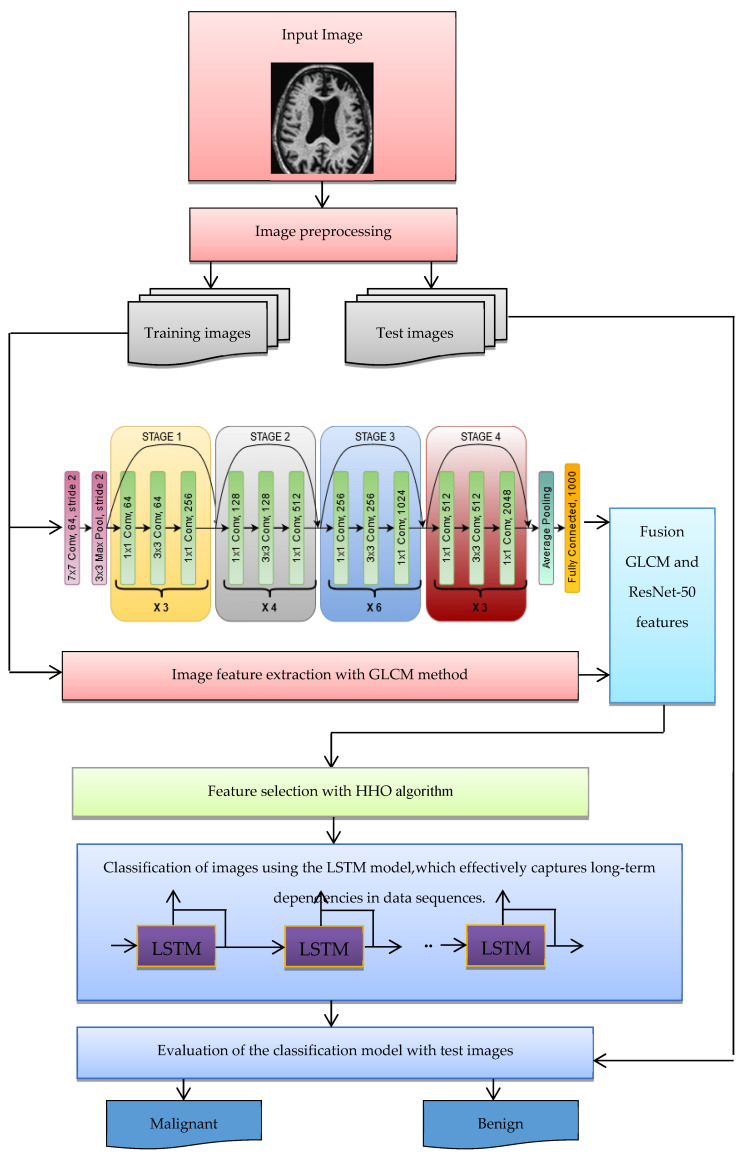
The structure of the suggested method for diagnosing Alzheimer’s disease.

The structure of the suggested approach for classifying malignant and benign Alzheimer’s images is shown in Figure 3. The following steps are used to categorize images into benign and malignant categories:

- Benign and malignant images are collected from datasets such as ADNI.
- The images of the dataset are pre-processed (possible noise is adjusted).
- The images are divided into two categories—training and test images.
- Extracting features of images using the ResNet-50 CNN architecture, which has shown exceptional performance in image classification tasks.
- Extracting image features with GLCM.
- Integration and combination of features extracted in the deep learning and GLCM stage.
- The HHO algorithm was selected over other optimization techniques due to its superior capability to balance exploration and exploitation, enabling a thorough search of the feature space while avoiding local optima. This characteristic is particularly crucial for high-dimensional data, such as the combined features extracted from ResNet-50 and GLCM in Alzheimer’s diagnosis. HHO’s adaptability has enabled it to effectively identify and retain the most relevant features while minimizing redundancy, thus enhancing the overall efficiency of the classification process. The algorithm’s robust and dynamic search mechanism ensures faster convergence, leading to improved model accuracy and reducing overfitting. These advantages position HHO as a more effective choice compared to other optimization methods, such as Genetic Algorithms (GA), Particle Swarm Optimization (PSO), and Simulated Annealing (SA), especially in addressing the complex requirements of feature selection and classification in medical image analysis.
- The optimal feature vector selected by the HHO algorithm is used for LSTM learning, ensuring high-classification computer performance.
- The purpose of the LSTM model is to categorize images into malignant and benign classes.
- With the test images, the trained LSTM model is evaluated for the classification of Alzheimer’s images. For evaluation, indices such as accuracy and classification error of Alzheimer’s images are used.

The following pseudocode (See Algorithm 1) provided outlines the process for classifying Alzheimer’s images as benign or malignant using a combination of techniques such as ResNet-50, GLCM, HHO, and LSTM.

**Algorithm 1** Alzheimer’s Image Classification using ResNet-50, GLCM, HHO, and LSTM 
1.
**Input Data:**
Dataset (ADNI3 containing benign and malignant Alzheimer’s images)Test images-ResNet-50 pre-trained modelGLCM (Gray Level Co-occurrence Matrix) methodHHO (Harris Hawks Optimization) algorithmLSTM (Long Short-Term Memory) model
2.
**Output:**
▪Classification results (malignant or benign)▪Performance metrics (accuracy, classification error)
3.**Step 1:** Load Dataset—Load benign and malignant Alzheimer’s images from the ADNI3 dataset4.**Step 2:** Preprocess Images—Perform preprocessing on the images (e.g., noise removal, normalization)5.**Step 3:** Split Dataset—Split dataset into training and testing images6.**Step 4:** Feature Extraction using ResNet-50—For each image, apply the ResNet-50 model to extract deep learning features7.**Step 5:** Feature Extraction using GLCM—For each image, apply GLCM to extract texture features8.**Step 6:** Combine Extracted Features—Integrate the features obtained from ResNet-50 and GLCM into a single feature vector9.**Step 7:** Feature Selection using HHO—Apply the HHO algorithm to select the optimal feature subset for Alzheimer’s classification10.**Step 8:** Train LSTM Model—Use the selected optimal features to train an LSTM model for classification11.**Step 9:** Classify Test Images—Apply the trained LSTM model to classify test images into malignant or benign classes12.**Step 10:** Evaluate Model Performance—Calculate and report performance metrics such as accuracy and classification error13.
**Return:**
Final classification results (malignant or benign)Performance metrics (accuracy, classification error)



### 3.2. Feature Extraction

A CNN is a feedforward neural network that, for some of its features, applies the convolution operation, although most of these networks have a deep architecture [41]. CNNs have been highly successful in processing multi-dimensional data, such as images, which places them among the most well-known types of DL algorithms [42]. The normal structure includes several convolutional, pooling, and fully connected layers, sometimes augmented with activation and dropout layers to improve generalization of the model and avoid overfitting. Convolutional layers take in input data and perform operations using learned kernels, forming feature maps by moving localized receptive fields across the data volume. In the feature map, each unit connects to a local region in the previous layer using weights and an activation’s function. The output ($x_{j}^{l}$) of the feature map l in the convolutional layer j can be obtained with Equation (1).

(1) $$x_{j}^{l}=f(\sum_{i\in M_{j}}x_{i}^{l-1}*k_{ij}^{l}+b_{j}^{l})$$

In this model, $k_{ij}^{l}$ represents the weight matrix of the convolution kernel j and $b_{j}^{l}$ represents the bias matrix associated with it. Mj represents the set of feature maps, * is the convolution operator, and f is the activation function.

(2) $$x_{j}^{l}={f(\beta_{j}^{l}down(x}_{i}^{l-1})+b_{j}^{l})$$

where ${down(x}_{i}^{l-1})$ is the maximum downsampling function, while $\beta_{j}^{l}$ and $b_{j}^{l}$ the multiplicative and additive biases, respectively.

**Figure 4:**
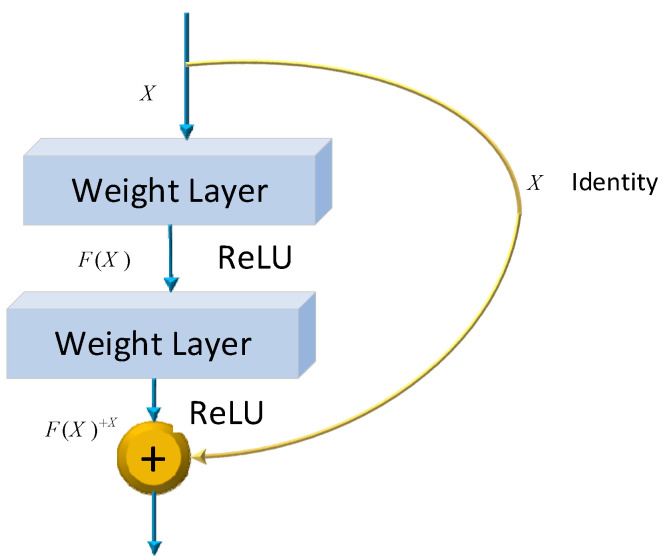
ResNet-50 residual block.

Downscaling the convolutional layer output reduces feature map dimensionality and total parameters, thereby enhancing the model’s translation invariance and robustness. The maximum pooling operation in layer l and the fully connected layer output $x^{l}$ are described in Equation (3).

(3) $$x^{l}=f(w^{l}x^{l-1}+b^{l})$$

where $w^{l}$ and $b^{l}$ be the weights and biases of the network, respectively. Several models have already been developed using the architecture of CNNs. One of the most famous ones is the ResNet architecture. Microsoft first introduced it in 2015. Since its development, many researchers have studied ResNet due to the simplicity of the architecture, fewer parameters, and the tremendous impact of performance on the model. Therefore, ResNet is related to images, such as image classification or image recognition everywhere. The ResNet-50 model represents an extension of the power of regular CNN by adding residual blocks connected by “shortcut connections”. In modeling, the architecture will ensure a nonlinear activation function, such as ReLU, to ensure smooth training and prevent problems like vanishing and center-exploding gradients. The structure of residual blocks in the ResNet-50 model is displayed in Figure 4.

**Figure 5:**
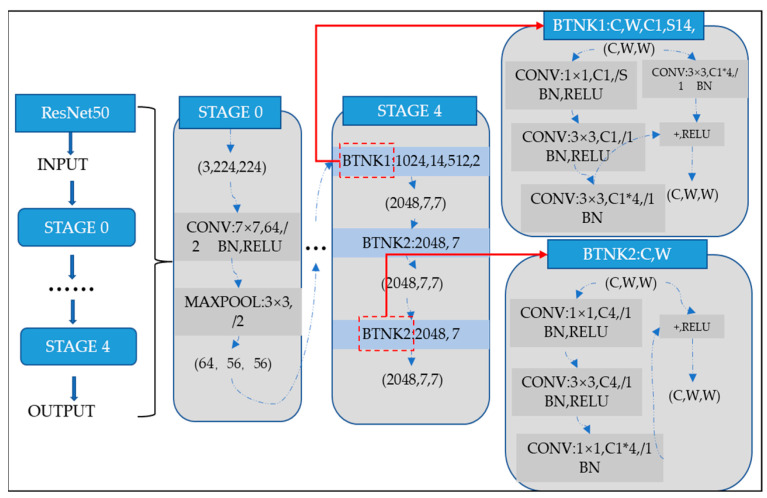
ResNet-50 neural network configuration.

ResNet-50 incorporates residual blocks in order to effectively avoid the vanishing and exploding gradient problems which emerge when deeper networks are built up. Furthermore, it can preserve more information in training for integrating the primary nonlinear features with the learned features from residual blocks. Due to its comprehensive parameters and high performance, ResNet-50 has been one of the most popular CNN models in recent years. Therefore, the classification of images of Alzheimer’s in this article is performed using the ResNet-50 model. The overall architecture of a ResNet-50 neural network is shown in Figure 5.

In this proposed system, the DL method of CNN is first applied during feature extraction. Then, further feature extractions from the images are done using techniques such as the Gray-Level Co-occurrence Matrix.

In the proposed framework, GLCM is employed as a feature extraction technique to enhance the model’s ability to analyze texture-based properties in medical images. GLCM is particularly effective in capturing the spatial relations between pixel intensities, which are crucial for identifying subtle textural patterns often associated with Alzheimer’s disease. By analyzing the co-occurrence of pixel intensities at specific distances and angles, GLCM generates a matrix that encapsulates key statistical measures including contrast, correlation, energy, and entropy. These measures provide a detailed representation of image textures, complementing the features extracted by DL methods like ResNet-50. The integration of GLCM into the feature extraction process enables the hybrid framework to leverage both deep learning’s high-level features and the texture-based insights from GLCM, resulting in improved classification accuracy and robustness.

Finally, the CNN and GLCM methods are combined to form a new set of features, which are then used for the feature selection phase.

In an image histogram, each radiance level is plotted against the number of pixels corresponding to that level, forming a graph of light intensity frequency. In contrast, GLCM analyzes image content by measuring how gray levels in neighboring pixels change or correlate with each other. GLCM constructs a matrix by examining pixel relationships at different angles and distances to enhance image features. Radius and angle are two parameters used to define the co-occurrence matrix of an image. The radius usually is taken as 1 or 2; the angles regarding eight adjacent pixels are taken for radius 1 as 0, 45, 90, 135, 180, 225, 270, and 315 degrees. In the GLCM technique, gray scales range from 0 to 255 for light intensity, while four particular angles are taken as 0, 45, 90, and 135 degrees. After developing the matrix, the measures of energy, contrast, correlation, and entropy are calculated using Equations (4)–(7) [43]:

(4) $$E=\sum_{i,j}{p(i,j)}^{2}$$

(5) $$C=\sum_{i,j}{\left|i-j\right|}^{2}\cdot p(i,j)$$

(6) $$H=\sum_{i,j}\frac{p(i,j)}{1+\left|i-j\right|}$$

In these equations, p (i,j) is the intensity of pixel light in row i and column j.

### 3.3. Feature Selection

In the new approach, every feature vector *i* in iteration *t* is represented by $X_{i}\left(t\right)$, and the population size or the total number of feature vectors in the HHO algorithm is represented as N. Based on this, in the algorithm, the best feature vector will be taken as X rabbit (t) which signifies the position of the prey. The average of those feature vectors is calculated by Equation (7), and the average is used in the equations of the HHO algorithm:

(7) $$X_{M}\left(t\right)=\frac{1}{N}\sum_{i=1}^{N}X_{i}\left(t\right)$$

where $X_{M}\left(t\right)$ is the centroid of the feature vectors. In the case of |E| ≥ 1, the feature vectors are updated randomly, following the behavior defined in Equation (8).

(8) $$X(t+1)=\left\{\begin{array}{c} \begin{array}{cc} X_{rand}\left(t\right)-r_{1}\left|X_{rand}\left(t\right)-2r_{2}\cdot X(t)\right| & \;\;\;\;\;\;\;\;\;\;\;\;\;\;\;\;\;\;\;rand\geq 0.5 \end{array}\\ \begin{array}{cc} {(X}_{rabbit}\left(t\right)-X_{M}\left(t\right))-r_{3}(LB+r_{4}(UB-LB)) & rand<0.5 \end{array} \end{array}\right.$$

X(t) indicates the current position of a feature vector in current iteration and X(t + 1) indicates its position in the next iteration. $X_{rand}\left(t\right)$ a feature vector that is randomly selected from the population. ${r_{1},r}_{2}$, $r_{3}$, and $r_{4}$ are random numbers between 0 and 1. LB and UB are the lower and upper bounds of each feature vector component, respectively.

Each feature vector has an associated energy level that governs the movement toward an optimal solution, formulated in Equation (9).

(9) $$E={2E}_{0}(1-\frac{t}{T})$$

where E is the energy coefficient that decreases randomly from 2 to 0. $E_{0}$ is the initial value of energy. It denotes the number of the current iteration. T represents the maximal number of iterations allowed to perform the metaheuristic. The HHO algorithm also updates the feature vectors with a Soft Besiege behavior, where the vectors will gradually converge toward the prey and start searching for the neighborhood to get a better solution. This behavior is modeled in Equations (10) and (11).

(10) $$X\left(t+1\right)=∆X\left(t\right)-E\left|J\cdot X_{rabbit}\left(t\right)-X\left(t\right)\right|$$

(11) $$∆X\left(t\right)=X_{rabbit}\left(t\right)-X\left(t\right)$$

In the search for quiet encirclement, the parameter J is a random value between zero and two, also called the prey’s escape step. Updating the feature vector using hard besiege is another behavior in the proposed method. In this behavior, the solutions or hawks dive towards the prey and try to reach a more optimal solution whose modeling is defined in Equation (12).

(12) $$X\left(t+1\right)=X_{rabbit}\left(t\right)-E\left|∆X\left(t\right)\right|$$

Updating the feature vector with quick dives is another behavior in the proposed method and is done according to Equation (13).

(13) $$X\left(t+1\right)=X_{rabbit}\left(t\right)-E\left|J\cdot X_{rabbit}\left(t\right)-X_{m}\left(t\right)\right|$$

By using these behaviors, the feature vectors can be updated in each iteration, and then the most optimal feature vector can be determined. The cost function is formulated to evaluate the feature vectors like Equation (14). In this function, two components of the average classification error of benign and malignant images and the average number of features selected as targets are formulated.

(14) $$Cost(X_{i})=w_{1}Error(X_{i})+w_{2}\frac{\left|n\right|}{\left|m\right|}$$

**Figure 6:**
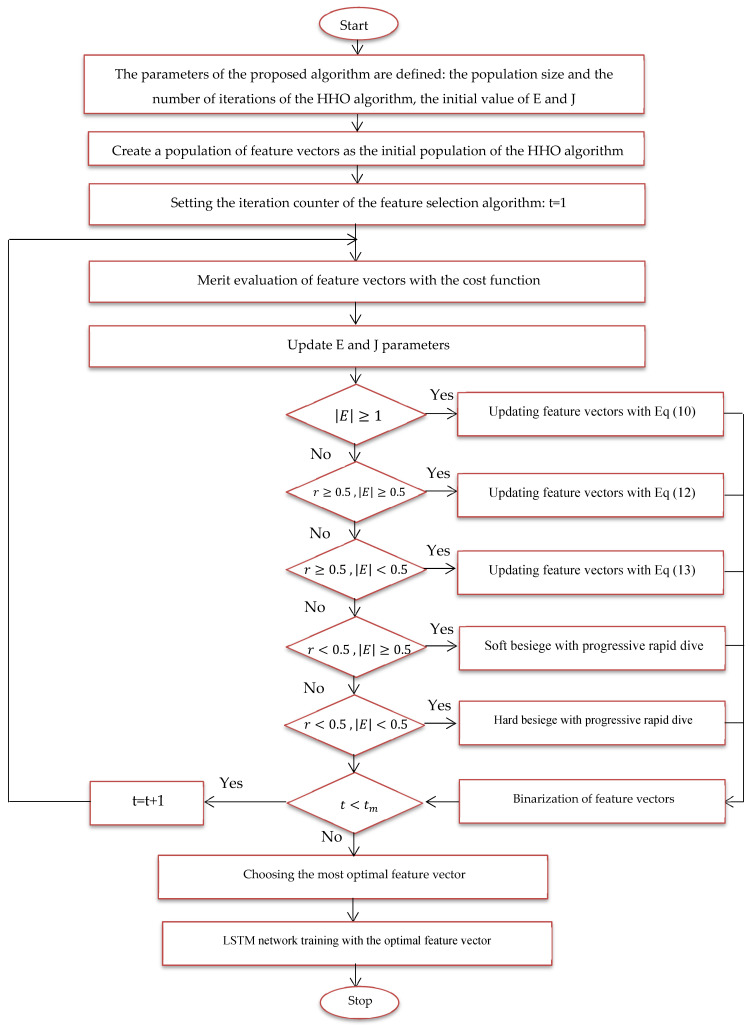
Proposed feature selection flowchart.

In this expression, Error ($X_{i}$) denotes the classification error for the Alzheimer’s images using the feature vector $X_{i}$, where |n| indicates the total number of dataset parameters and |m| signifies the number of parameters considered. Two weights, $w_{1}$ and $w_{2}$, are introduced into the equation to account for the error factor and the dimensionality reduction factor in the classification of malignant and benign images, respectively. The optimal feature vector for feature selection is defined at the end of the last iteration; Figure 6 provides a flowchart of the suggested model using the HHO algorithm.

- Size of training and test data for training and evaluation of the proposed method.
- A feature vector is considered and coded as a member of the HHO algorithm.
- Several feature vectors are considered problem solutions or members of the HHO algorithm, and this population is randomly generated.
- Solutions or hawks, which are the feature vector here, are evaluated with the cost function of the problem, and the most optimal feature vector is determined for image classification.
- Phases of random search, smooth encirclement, dive, etc. applied to feature vectors to be updated.
- By repeating the steps of the proposed algorithm, the most optimal feature vector is updated in each iteration.
- In the last iteration, the most optimal feature vector is extracted.
- The most optimal feature vector is used to train the LSTM network.
- The proposed method is tested with images in the feature selection phase.

### 3.4. Classification of Images with LSTM

**Figure 7:**
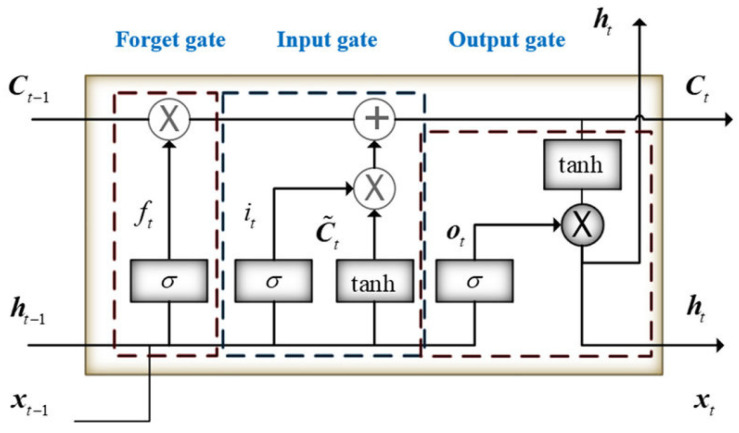
Structure of the LSTM.

Introduced by Hochreiter and Schmid Huber in 1997, LSTM is a model that enhances the performance of RNNs by employing a specific arrangement of hidden units and gates that control ’memory cells’ designed for long-term information storage. Although RNNs are straightforward and compact, they often struggle with learning long-term dependencies. In contrast, LSTMs are equipped with three essential gates: an input gate, a forget gate, and an output gate, which enable them to effectively learn these dependencies. This capability allows LSTMs to store information from previous time steps and modify the weights in the network at the current time step to accurately predict the next sequence step. LSTM features three gates: an input gate, a forget gate, and an output gate, as shown in Figure 7.

In the LSTM neural network, the forget gate is built to show how much of the previous memory it remembers and how much it has forgotten. For LSTM, the hidden state *ht* is calculated as Equations (15)–(19):

(15) $$f_{t}=\sigma (W_{f}\cdot X_{t}+U_{f}\cdot h_{t-1}+b_{f})$$

(16) $$O_{t}=\sigma (W_{o}\cdot X_{t}+U_{o}\cdot h_{t-1}+b_{0})$$

(17) $$\tilde{C_{t}}=tanh(W_{c}\cdot X_{t}+U_{c}\cdot h_{t-1}+b_{c})$$

(18) $$C_{t}=f_{t}\cdot C_{t-1}+i_{t}\cdot \tilde{C_{t}}$$

(19) $$h_{t}=tanh(C_{t})+O_{t}$$

## 4. Experiments

In this section, the proposed approach is implemented on a set of images of Alzheimer’s patients. Tensorflow and Keras libraries of Python have been used for implementation. The images used for evaluation are classified into malignant and benign categories. This part of the dataset presents evaluation criteria and comparisons to analyze the proposed approach.

### 4.1. Dataset

**Figure 8:**
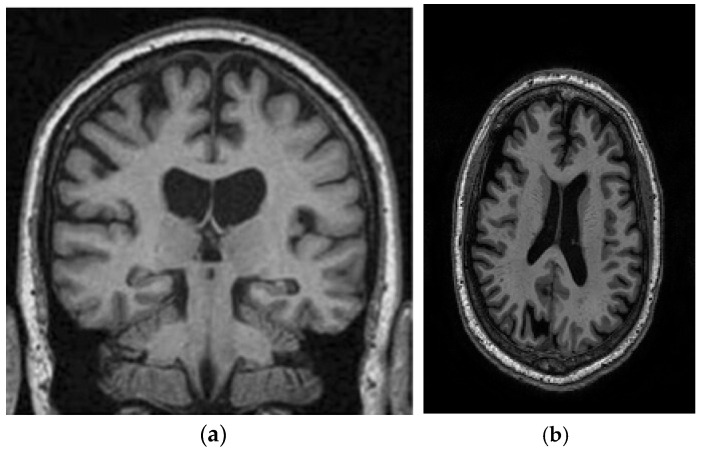
Two sample images from the ADNI and MIRIAD datasets: (**a**) MIRIAD dataset [46]; (**b**) slices from the ADNI Dataset [47].

This study utilizes two distinct datasets, ADNI and MIRIAD, to investigate Alzheimer’s disease, ensuring a diverse range of demographics and imaging modalities. The ADNI dataset, which tracks the early stages of Alzheimer’s, consists of MRI scans, specifically 128 sagittal slices per scan, each formatted as a 256 × 256 matrix. It includes a total of 741 participants, with 314 images from Alzheimer’s patients and 427 from normal controls. The participants represent a wide variety of ages, genders, and ethnic backgrounds, ensuring that the model is robust and can perform well across different populations. The MIRIAD dataset also focuses on Alzheimer’s, containing MRI brain scans from 46 Alzheimer’s patients and 23 normal controls, with follow-up scans taken at intervals ranging from 2 weeks to 2 years [44,45]. Both datasets are sourced from various institutions, introducing variability in the acquisition conditions, which further enhances the model’s robustness and its ability to generalize to real-world scenarios. The inclusion of different ethnic backgrounds, as well as varying patient ages and scan intervals, ensures that the model is adaptable to diverse clinical environments. Figure 8 shows examples of benign and malignant images from these datasets. Table 1 summarizes the key settings and configurations used in the experiments to analyze Alzheimer’s disease using HHO and CNN-based approaches.

To ensure consistent input for model training and enhanced data quality, several preprocessing steps were applied to the MRI images in both the ADNI and MIRIAD datasets. Initially, noise reduction was performed using a Gaussian smoothing filter. MRI images, particularly in clinical settings, are often impacted by noise, including thermal and motion-induced artifacts. Applying Gaussian smoothing helps preserve crucial structural features while minimizing unwanted noise, enabling the model to focus on relevant patterns rather than distractions that could compromise its performance. Subsequently, skull removal was performed using a skull-stripping algorithm, such as the Brain Extraction Tool (BET), which is commonly used in neuroimaging. This step eliminates non-brain tissue, such as skull, scalp, and surrounding tissues, from MRI scans, leaving only the brain matter for analysis. Skull removal is essential in reducing computational complexity and allowing the model to focus on the brain areas critical for Alzheimer’s detection. Without this step, the presence of non-brain tissue could hinder feature extraction and affect classification accuracy.

These preprocessing techniques were chosen to enhance the model’s ability to detect subtle patterns in brain structure that may indicate Alzheimer’s disease while minimizing the influence of irrelevant features and noise. By concentrating on clean, brain-only images, the model can more effectively learn from the underlying anatomical features, leading to improved generalization and performance.

### 4.2. Evaluation Indexes

Accuracy, sensitivity, and precision indices are used to classify Alzheimer’s images, and the equation of each of these indices is presented in Equations (20)–(22):

(20) $$Acc=\frac{TP+TN}{TP+TN+FP+FN}\times 100\%$$

(21) $$Recall=\frac{TP}{TP+FN}\times 100\%$$

(22) $$precision=\frac{TN}{TN+FP}\times 100\%$$

### 4.3. Result and Analysis

**Figure 9:**
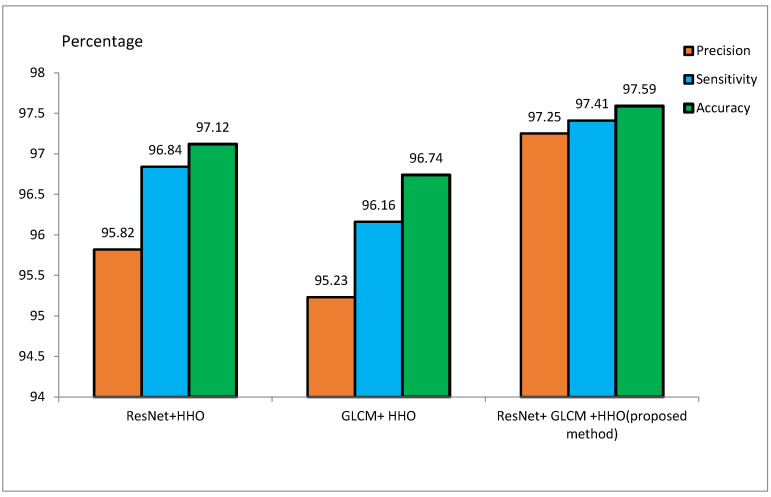
Evaluation of the proposed method on the ADNI dataset.

**Figure 10:**
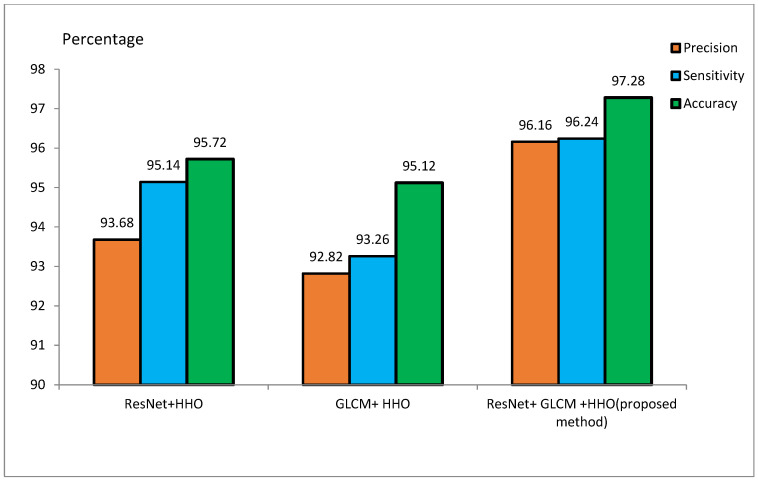
Evaluation of the proposed method on the MIRIAD dataset.

The MRI dataset is pre-processed to match the CNN input size. It involves the extraction of the brain from 3D MRI scans, skull removal, and the reduction of image noise for better model efficiency. Smoothing of MRI images reduces the noisiness and lowers the resolution. Smoothing is done by a 4 mm FWHM Gaussian filter, where FWHM stands for the width of the kernel. Since the ResNet architecture requires 224 × 224 pixel images, every MRI image is resized to these dimensions before feeding it to the model. For the training of the model, 70% of the samples are to be used for training, and the remaining 30% are to be used for testing purposes. During the implementation, 20 feature vectors were used, and the HHO algorithm was iterated for 100 runs. Initial values of *E* and *J* were set to 2. Table 2 and Table 3 represent the accuracy, sensitivity, and precision of the suggested approach for the ADNI and MIRIAD datasets, respectively, for different scenarios. Figure 9 and Figure 10 represent the performance metrics of the proposed approach for the same datasets.

To achieve effective classification, the study employed two distinct feature extraction methods, ResNet and GLCM, and their details are provided below.

- ResNet Features: The ResNet model extracts a feature vector from input MRI images following pre-processing steps, such as resizing the images to 224 × 224 pixels. In this experiment, 20 features were extracted and used for classification.
- GLCM Features: GLCM is an additional feature extraction method used in this study. While ResNet captures DL-based features, GLCM focuses on texture-based features. A total of 20 features were selected from GLCM for classification, in line with the experimental design.
- Combined feature set: Features derived from ResNet and GLCM were merged for a comprehensive feature set for classification. Since each technique contributed 20 features, the combined feature set comprised 40 features in total (20 from ResNet and 20 from GLCM).

**Feature Counts:**

- ResNet Features: 20
- GLCM Features: 20
- Combined Feature Set: 40 (20 + 20)

Precision, sensitivity, and accuracy are key metrics for evaluating model performance in medical imaging, especially in Alzheimer’s diagnosis using MRI scans. Precision measures the proportion of true positives (correctly identified Alzheimer’s patients) out of all positive predictions, helping avoid misdiagnosis of healthy individuals as patients. Sensitivity (or recall) calculates the proportion of true positives out of all actual positive cases, ensuring that all Alzheimer’s patients are correctly identified and minimizing missed diagnoses. Accuracy provides an overall assessment of the model’s ability to classify both Alzheimer’s patients and healthy individuals correctly. However, in imbalanced datasets, where healthy individuals outnumber Alzheimer’s patients, accuracy alone can be misleading. Combining accuracy with precision and sensitivity provides a more comprehensive evaluation of model performance.

In this study, these metrics are crucial for ensuring reliable predictions and accurate diagnoses that directly impact patient outcomes. The use of a hybrid approach with features from ResNet and GLCM aims to optimize these metrics across various imaging modalities and patient demographics, enhancing the model’s robustness.

#### 4.3.1. Performance Overview

Table 2 presents performance metrics of feature extraction methods using ResNet and GLCM, both stand-alone and combined with the HHO algorithm. The experiments conducted on the ADNI dataset reveal that the ResNet + HHO method provides accuracy, sensitivity, and precision values of 97.12%, 96.84%, and 95.82%, respectively. For GLCM+HHO, these measures are 96.74%, 96.16%, and 95.23%, respectively. Therefore, the suggested approach developed founded on the integration of the two methodologies yields an accuracy as high as 97.59%, a sensitivity as high as 97.41%, and a precision as high as 97.25% using the ADNI dataset.

#### 4.3.2. Performance on the MIRIAD Dataset

Table 3 presents ResNet and GLCM feature extraction alone and combined with the HHO algorithm for feature optimization on MIRIAD. The ResNet+HHO method classified malignant and benign images from Alzheimer’s disease with an accuracy, sensitivity, and precision rate of 95.72%, 95.14%, and 93.68%, respectively. In comparison, the GLCM+HHO method returned accuracy values of 95.12%, with 93.26% sensitivity and 92.82% precision. The proposed method of ResNet and GLCM feature extraction with HHO feature selection had better performance in accuracy, sensitivity, and precision, the values being 97.28%, 96.24%, and 96.16%, respectively. This study concludes that ResNet may have better diagnostic accuracy for classifying Alzheimer’s disease than GLCM. A combination of ResNet and GLCM, feature extraction methodologies, contributed a lot to the improved overall accuracy of the proposed method. The image texture-based GLCM method improves classification accuracy when combined with DL techniques using CNN. The efficiency of the proposed method on the ADNI dataset is in contrast with the techniques outlined in [48].

The proposed method combines two feature extraction techniques—ResNet and GLCM—benefiting from ResNet’s ability to capture complex patterns in MRI images and GLCM’s focus on fine-grained texture features. This hybrid approach improves Alzheimer’s disease classification accuracy, sensitivity, and precision. Additionally, the HHO algorithm is used for feature selection, reducing overfitting and improving generalization.

**Figure 11:**
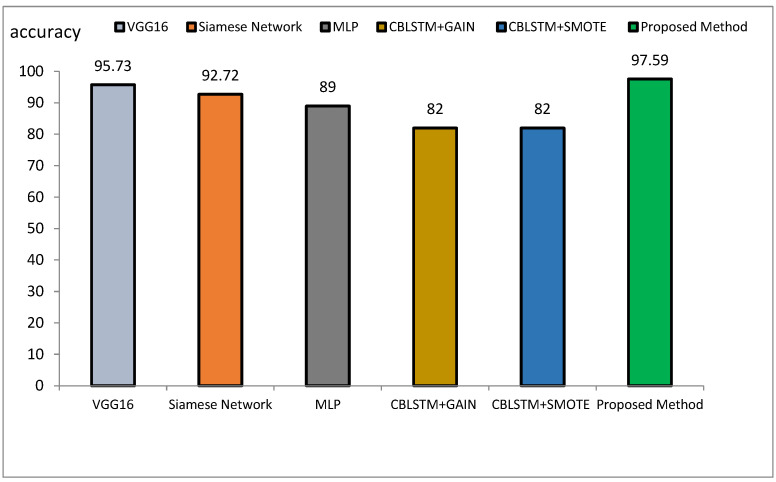
Comparison of the proposed method with several classification methods of Alzheimer’s images with accuracy index.

**Figure 12:**
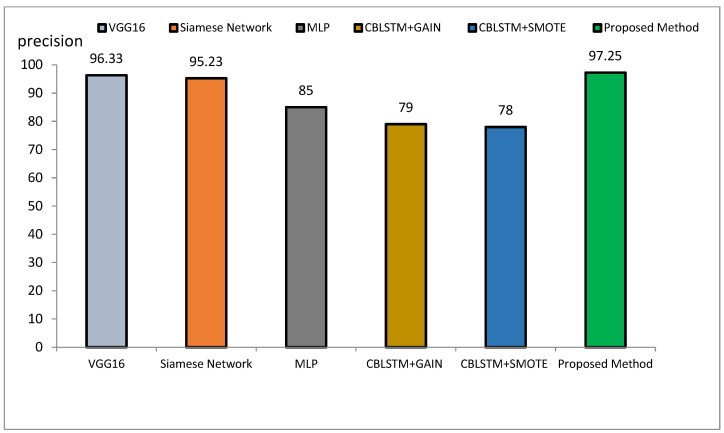
Comparison of the proposed method with several classification methods of Alzheimer’s images with precision index.

**Figure 13:**
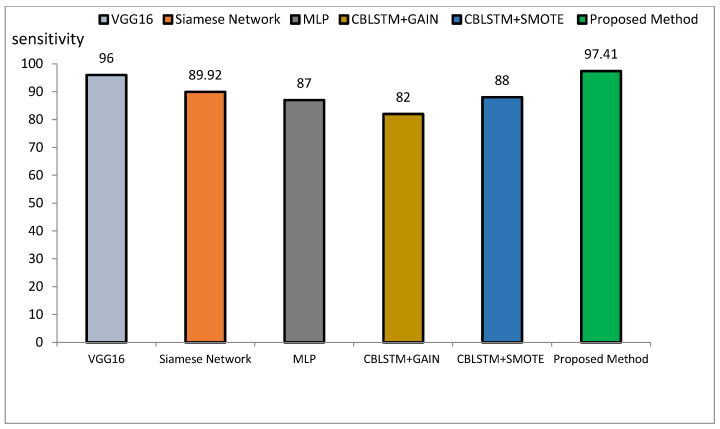
Comparison of the proposed method with several classification methods of Alzheimer’s images with sensitivity index.

The method proposed outperforms other state-of-the-art models, such as VGG16, Siamese Networks, MLP, and CBLSTM+GAIN, particularly in capturing specialized texture features important for Alzheimer’s diagnosis. It also demonstrates superior generalization across different datasets, making it more robust and reliable for clinical applications. The integration of ResNet and GLCM with HHO optimization results in a more accurate, sensitive, and precise model, making it highly suitable for medical imaging tasks requiring high accuracy and reliability. The comparative performance of the proposed method against VGG16, Siamese Network, MLP, CBLSTM+GAIN, and CBLSTM+SMOTE is presented in terms of accuracy, precision, and sensitivity in Figure 11, Figure 12 and Figure 13, respectively.

**Figure 14:**
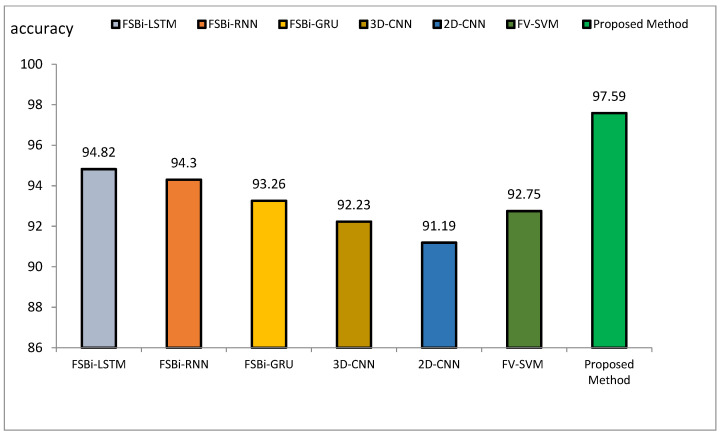
Comparison of the accuracy of the proposed method of DL methods in Alzheimer’s diagnosis.

**Figure 15:**
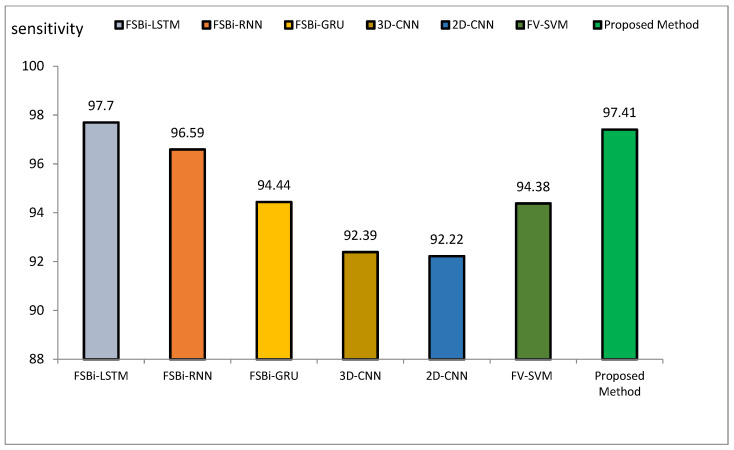
Comparison of the sensitivity of the proposed method of DL methods in Alzheimer’s diagnosis.

The accuracy rate for VGG16, Siamese Network, MLP, CBLSTM+GAIN, and CBLSTM+SMOTE is 95.73%, 92.72%, 89%, 82%, and 82%, respectively. The proposed method’s accuracy is higher than those discussed in [48,49] for diagnosing Alzheimer’s disease, as illustrated in Figure 14 and Figure 15. Among the mentioned methods, VGG16 performed strongly but still less well than the new approach. The outline showed that the proposed method outperformed VGG16, Siamese Network, MLP, CBLSTM+GAIN, and CBLSTM+SMOTE regarding accuracy, sensitivity, and precision. It performs better because it incorporates CNN and GLCM architectures for feature extraction.

The accuracy index of FSBi-LSTM, FSBi-RNN, FSBi-GRU, 3D-CNN, 2D-CNN, and FV-SVM is 94.82%, 94.3%, 93.26%, 92.23%, 91.19%, and 92.75%, respectively. The accuracy index of the proposed method for classifying Alzheimer’s patients is 97.59%. The new approach is more accurate than these methods. The sensitivity index of the compared methods is 97.7%, 96.59%, 94.44%, 92.39%, 92.22%, and 94.38%, respectively. Among the compared methods, the FSBI-LSTM method has more sensitivity than others, but the proposed method has more accuracy than FSBI-LSTM.

## 5. Discussion

The findings of this research emphasize the efficiency of the new hybrid framework, which integrates ResNet, GLCM, and HHO for feature extraction and selection, along with LSTM for image categorization. The effectiveness of the proposed approach was tested on two gold-standard databases, ADNI and MIRIAD, and demonstrated superior accuracy, sensitivity, and precision compared to other approaches.

The results obtained with the new method demonstrated an accuracy of 97.59%, sensitivity of 97.41%, and precision of 97.25% on the ADNI dataset, showing significantly better performance than other methods like ResNet+HHO and GLCM+HHO, which produced lower metrics. On the MIRIAD dataset, similar outcomes were observed, with the new approach achieving an accuracy of 97.28%, sensitivity of 96.24%, and precision of 96.16%. These results indicate that combining ResNet and GLCM with HHO as an optimization algorithm enables the extraction of more classification-relevant features. To demonstrate the robustness of the results compared to other methods, statistical tests such as *p*-values can be included. These tests will help in quantifying the significance of the performance differences between the proposed method and others, such as ResNet+HHO and GLCM+HHO. For example, a paired *t*-test or ANOVA could be used to compare the accuracy, sensitivity, and precision metrics obtained with the new method against those obtained from the other methods. A *p*-value less than 0.05 would indicate that the observed differences are statistically significant and not due to random chance. Furthermore, confidence intervals for each metric can further strengthen the conclusions regarding the reliability and consistency of the proposed method’s performance.

The hybrid model consistently outperformed all evaluation metrics when comparing the proposed approach with other DL models such as VGG16, Siamese Network, MLP, CBLSTM+GAIN, and CBLSTM+SMOTE. For example, the proposed method’s accuracy of 97.59% exceeded that of VGG16 at 95.73%, further emphasizing its robustness. The integration of CNN and GLCM in the feature extraction phase, along with HHO for feature selection, contributed significantly to the model’s high performance, enhancing its ability to distinguish between benign and malignant Alzheimer’s images.

The improvement in accuracy, sensitivity, and precision can be attributed to the multi-stage feature extraction process, which combines texture-based analysis through GLCM with the DL capabilities of CNNs. Additionally, the HHO algorithm effectively selects the most relevant features, reducing the dimensionality of the data and improving the model’s efficiency. This combination allowed the LSTM network to learn more representative patterns from the images, leading to better classification results. To address the limitations of the proposed method, such as increased computational complexity and longer training times, several strategies can be employed. One approach is to optimize the model architecture by reducing the number of parameters in the ResNet and GLCM stages while maintaining classification accuracy. Moreover, techniques such as transfer learning could be explored in order to leverage pre-trained models, which can significantly reduce the training time and computational resources required. Another possibility is using parallel or distributed computing systems that accelerate training, making the process more efficient. Furthermore, applying pruning or quantization methods to the model after training could reduce its size and improve inference speed, without compromising performance. These approaches can help mitigate the computational challenges while still benefiting from the high accuracy and reliability offered by the proposed method. One limitation of the proposed method, however, is its increased complexity and training time compared to simpler models such as ResNet-50 alone. The addition of the GLCM and HHO stages increases the computational burden, making the method more resource-intensive. Nonetheless, the significant improvement in classification accuracy justifies the added complexity, especially in critical applications such as early Alzheimer’s disease detection, where accurate and reliable diagnoses are paramount.

The proposed method, combining ResNet, GLCM, and HHO, can be adapted to other neurological conditions such as brain tumors by capturing both high-level patterns (ResNet) and texture details (GLCM) from MRI or CT scans, improving the classification performance. The HHO-based feature selection enhances model generalization. Integrating Generative Adversarial Networks (GANs) could further boost performance by generating synthetic data for training, addressing class imbalance, and enhancing robustness. GANs could also create varied data to improve classification of other conditions like stroke or epilepsy. To optimize training time, more efficient feature selection methods (e.g., recursive feature elimination, PCA) and techniques like knowledge distillation or transfer learning could be explored.

Future research could explore applying this hybrid approach to other neurological disorders, such as brain tumors, to assess its generalizability. Additionally, advanced DL networks like GANs could be integrated to improve the model’s performance further. Optimizing the training time without sacrificing accuracy could also be an area of focus, potentially by exploring more efficient feature selection methods.

The proposed hybrid method combining ResNet, GLCM, HHO, and LSTM offers a promising solution for classifying Alzheimer’s disease images, achieving superior accuracy, sensitivity, and precision compared to other state-of-the-art models. This approach has significant potential for improving diagnostic tools in medical imaging applications, providing a more accurate and reliable method for early Alzheimer’s disease detection.

## 6. Conclusions

Alzheimer’s disease causes many deaths every year, and early detection is essential to increase the survival rate of patients. Early detection based on medical imaging is one of the effective methods for early detection of Alzheimer’s disease. The classification of images into benign and malignant classes of Alzheimer’s, depending on the CT scans, assists in assessing the severity of the disease. Most research works have employed deep learning-based methodologies for feature extraction and selection.

The proposed detection of Alzheimer’s disease in this paper is based on medical images that apply a multi-step procedure. It begins by extracting features from the images using ResNet-50 and the GLCM method. After that, the HHO algorithm is applied to feature selection. For the classification stage, DL methods and an LSTM neural network are applied to distinguish between benign and malignant images. The process, implemented in Python on two datasets, namely ADNI and MIRIAD, resulted in the following performance: ADNI—97.59% accuracy, 97.41% sensitivity, and 97.25% precision; MIRIAD—97.28% accuracy, 96.24% sensitivity, and 96.16% precision.

The proposed method is also much better in terms of accuracy than other methods (VGG16, Siamese Network, MLP, CBLSTM+GAIN, and CBLSTM+SMOTE), since its results for image classification had higher accuracy in FSBi-LSTM, FSBi-RNN, FSBi-GRU, 3D-CNN, 2D-CNN, and FV-SVM. Though the proposed approach had higher accuracy compared with other DL approaches such as CNN, RNN, GRU, and LSTM, it has more complexity and requires more time to train compared with ResNet-50. This architecture will be applied in future research in other brain-related conditions, such as brain tumors, and in studying advanced DL networks, including GANs, for the diagnosis of Alzheimer’s.

## Figures and Tables

**Table 1:** Experimental parameters for HHO- and CNN-based approaches.

Parameter	Value
Algorithm	HHO
Dataset	ADNI, MIRIAD
MRI Image Dimensions	256 × 256 (pre-processed to 224 × 224 for CNN)
Pre-processing Steps	Skull removal, noise reduction, smoothing (4 mm FWHM Gaussian filter)
Feature Extraction Method	ResNet, GLCM
Optimization Algorithm	HHO
Number of Features Used	20
Number of Iterations (HHO)	100
Population Size (HHO)	30
Initial Values of E and J	2
Training Set Percentage	70%
Testing Set Percentage	30%
Evaluation Metrics	Precision, Sensitivity, Accuracy
Libraries Used	TensorFlow, Keras (Python)

**Table 2:** Precision, sensitivity, and precision of the new approach in the ADNI dataset.

Methods	Precision	Sensitivity	Accuracy
ResNet + HHO	95.82	96.84	97.12
GLCM + HHO	95.23	96.16	96.74
ResNet + GLCM + HHO (proposed method)	97.25	97.41	97.59

**Table 3:** Precision, sensitivity, and accuracy values of the new approach in the MIRIAD dataset.

Methods	Precision	Sensitivity	Accuracy
ResNet + HHO	93.68	95.14	95.72
GLCM + HHO	92.82	93.26	95.12
ResNet + GLCM + HHO (proposed method)	96.16	96.24	97.28

## Data Availability

The datasets generated and analyzed during the current study are available from the corresponding author on reasonable request.
